# Influence of foot arch height on isokinetic knee muscle strength at varying angular velocities

**DOI:** 10.3389/fspor.2026.1616379

**Published:** 2026-03-24

**Authors:** Jinqun Ye, Shuang Guo, Feisheng Zheng, Weibang Gao, Bo Wu, Jinze Tan, Yunping Zhang, Guangqing Xu, Lei Zhang

**Affiliations:** 1 Department of Rehabilitation Medicine, Guangdong Provincial People's Hospital, Guangdong Academy of Medical Sciences, Southern Medical University, Guangzhou, China; 2 Clinical Medical College of Acupuncture Moxibustion and Rehabilitation, Guangzhou University of Chinese Medicine, Guangzhou, China

**Keywords:** foot arch height, isokinetic moment, isokinetic muscle strength test, muscle strength, plantar pressure test

## Abstract

**Background:**

The height of the foot arch is highly related to knee joint function. Knee joint strength is a significant contributing factor to knee joint injuries, while existing research has not yet clearly established the correlation between arch height and knee joint strength.

**Objective:**

This study aims to investigate the influence of arch height on isokinetic knee muscle strength at varying angular velocities, thereby providing a potential scientific basis for the prevention and rehabilitation of associated conditions.

**Design:**

Cross-sectional study.

**Participants:**

Sixty healthy young adults (21, 22) years old were categorized into three groups based on arch classification (10 male and 10 female per group): low arch (LA; height 1.67 ± 0.075 m, weight 65.60 ± 13.11 kg), normal arch (NA; height 1.67 ± 0.084 m, weight 60.20 ± 9.22 kg), and high arch (HA; height 1.69 ± 0.080 m, weight 58.95 ± 6.60 kg).

**Methods:**

Arch classification was determined via the arch index (AI) using a plantar pressure measurement analyzer. Knee flexor and extensor muscle strength were assessed at slow (60 °/s) and fast (180 °/s) velocities using the ISOMED2000 isokinetic muscle strength testing system. Isokinetic moment curves (IMC) from 10° to 90° were recorded and analyzed statistically.

**Results:**

At 60 °/s, compared with NA, LA exhibited higher knee extension (55–83°) and flexion (73°–90°) moments, while flexion moments were lower in the 31–58°range (*P* < 0.05). In contrast, HA exhibited lower knee extension (20°–66°) and flexion (43°–65°and 74°–86°) moments (*P* < 0.05). At 180 °/s, both LA and HA groups had significantly lower knee extension (LA: 26°–90°; HA: 14°–90°) and flexion moments (LA: 10°–80°; HA: 10°–90°) compared with NA (*P* < 0.05).

**Conclusion:**

This study demonstrates that foot arch height is associated with knee muscle strength at varying angular velocities. LA individuals exhibited greater muscle strength in the posterior segments of the knee range at lower velocities, while those with abnormal arch heights (LA and HA) demonstrated weaker knee muscle strength at high velocities. These findings suggest that individuals with abnormal arch heights may benefit from incorporating targeted high-speed knee exercise training to enhance muscle performance and mitigate injury risk in competitive sports.

## 1 Introduction

Foot arch height is generally classified into three types based on the morphology of the medial longitudinal arch: low arch (LA), normal arch (NA), and high arch (HA) (1). LA and HA are common foot morphological deviations in adults, with prevalence rates of 2%–23% for LA (2) and 10% for HA (3). Abnormal arch height alters knee kinematics (4), with low arches associated with heel valgus and tibial internal rotation (2, 5), and high arches linked to heel varus and tibial external rotation (6, 7).

Abnormal foot arch height is a significant mechanical factor influencing knee joint function during movement (4). Its effects manifest across multiple domains: static balance (8, 9), knee joint position sense (10), dynamic loading and control (where it disrupts knee joint motor control mechanisms (11), alters frontal plane kinetics (12), and increases medial tibiofemoral joint loading (13).The ultimate consequence of these pathological mechanical alterations is a significantly elevated risk of knee joint injury and degeneration, as demonstrated in studies on anterior cruciate ligament (ACL) injury (14) and the symptoms and characteristics of knee osteoarthritis (15, 16). These findings collectively establish aberrant foot arch morphology as a key risk factor (12, 17).

Knee muscle strength, as the mechanical foundation of joint stability, is directly associated with an increased risk of injury (18, 19), as well as pain and reduced quality of life (20) in patients with knee osteoarthritis (KOA). Foot arch height, as the initiating point of lower limb kinetics, is hypothesized to influence knee muscle strength (21–23); however, direct evidence remains inconsistent. For instance, Bakırhan et al. (24) found no significant association using manual muscle testing, suggesting potential obscuration of a true effect by methodological limitations. Conversely, Kim et al. (25) reported that the impact of foot arch height on knee joint moments was detectable only during walking, implying that movement velocity may serve as a critical moderating variable. Thus, moving beyond the limitations of manual testing (ambiguity) and speed-specific observations (constraints), a systematic investigation into the quantitative effects of foot arch height on knee muscle strength across varying movement velocities is essential to elucidate the underlying biomechanical mechanisms.

Isokinetic muscle strength testing represents a sophisticated method for quantitatively assessing muscle strength across the full range of motion of a joint at a constant velocity (26, 27). This approach is characterized by high efficiency (28) and reliability (29), and precisely delineates force variations throughout the range of motion—as evidenced by isokinetic moment curves (IMCs) that depict the relationship between moment and angle, curve morphology, and smoothness (30). Isokinetic muscle strength testing serves both diagnostic and screening purposes for musculoskeletal injuries of lower extremity (31), as moments measured at different angular velocities carry distinct clinical significance: ≤60 °/s measurements assess muscular strength magnitude, while ≥180 °/s measurements evaluate power or endurance (32). A meticulous analysis of IMC facilitates a nuanced understanding of muscle function at various angles and velocities, providing valuable insights into muscle performance and potential deficits. Therefore, the aim of this study was to elucidate the effect of foot arch height on knee muscle function by comparing knee IMCs among subjects with low, normal, and high arch profiles under varying angular velocities. We hypothesize that individuals with LA and HA would demonstrate distinct extension and flexion moments compared to NA controls at varying angular velocities. Elucidating these biomechanical variations holds dual clinical relevance: (1) establishing a theoretical framework for knee injury prevention and rehabilitation strategies, and (2) advancing the mechanistic understanding of foot arch height's role in lower limb kinetics.

## 2 Methods

### 2.1 Participants and study design

The sample size was determined based on Cohen's effect size classification (33), with a large effect size (Cohen's *f* = 4.5) selected for estimation. Power analysis was conducted using G*Power 3.1 (34) software (one-way ANOVA, *α* = 0.05, power = 1 − *β* = 0.80, three groups), yielding a required sample size of 17 participants per group (total *N* = 51). Accounting for an anticipated 15% attrition rate, we planned to recruit 60 participants (20 per group). A total of 73 healthy participants were initially enrolled in the study between September 5, 2022, and September 12, 2024. After excluding 13 participants due to invalid test results, 60 eligible participants (30 males and 30 females) were included in the final analysis. The study was approved by the Medical Ethics Review Committee of Guangdong Provincial People's Hospital (KY2023-788-02). All subjects provided written informed consent after receiving comprehensive information about the study protocol.

Inclusion criteria were as follows: (1) age 20–30 years, with no gender restrictions and no pregnancy; (2) consistent bilateral foot shape meeting AI criteria.

Exclusion criteria were as follows: (1) restricted knee joint movement; (2) chronic lower limb diseases (e.g., osteoarthritis); (3) use of medications that could affect assessment results (e.g., analgesics); (4) congenital or acquired foot/knee deformities confirmed by clinical orthopedic examination; (5) history of lower extremity injury, surgery or fracture; (6) autoimmune or neurological disease; (7) regular physical activity habits (<150 min/week of moderate-intensity or <75 min/week vigorous-intensity aerobic activity, or suboptimal combined equivalents) (35); (8) history of orthopedic shoes or medical therapeutic footwear use.

**Figure 1:**
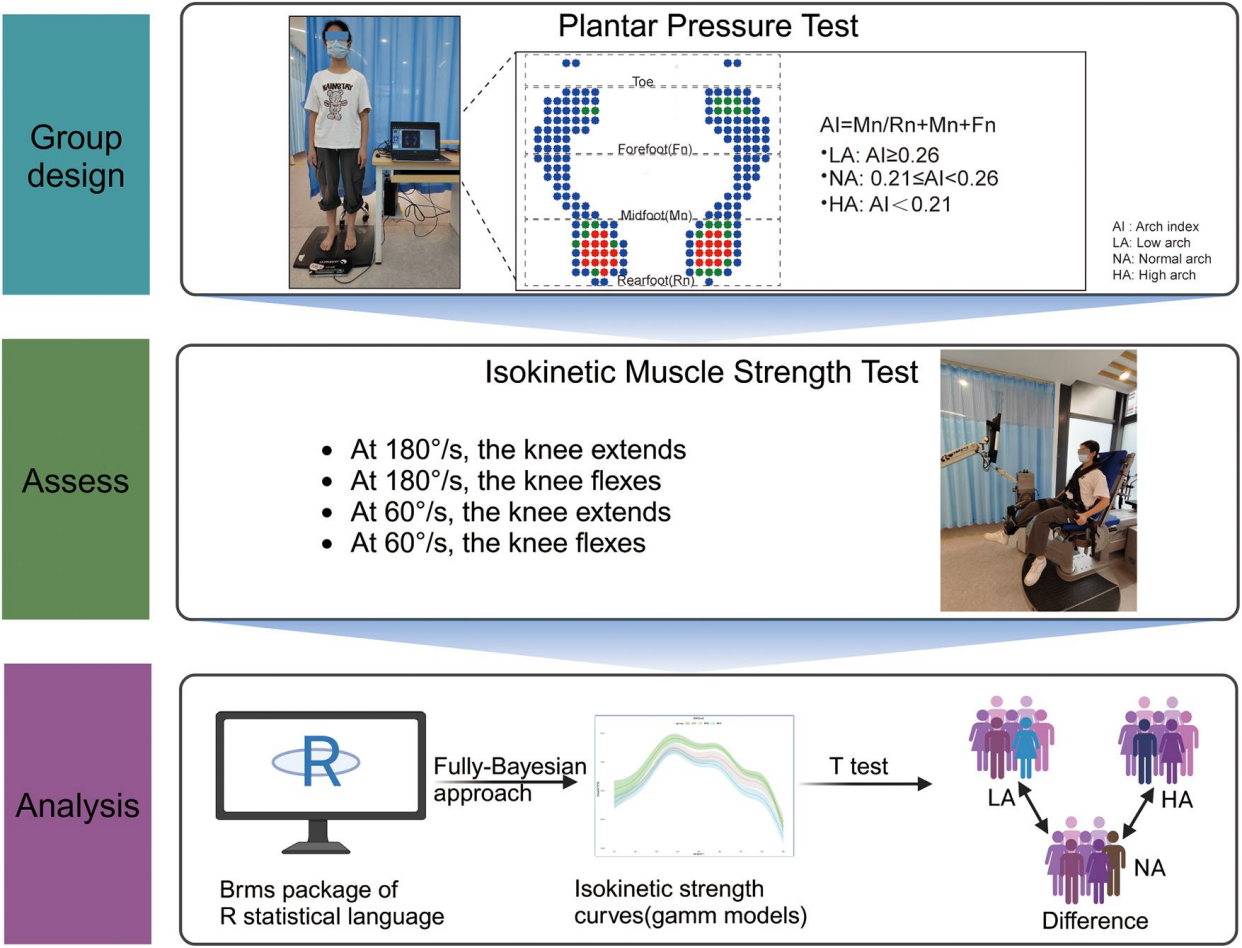
Study design. Healthy volunteers were stratified into three groups based on plantar pressure assessment via the arch index (AI). Subsequently, these groups underwent isokinetic muscle strength testing at two distinct angular velocities (60 °/s and 180 °/s). The isokinetic moment curves (IMC) were modeled using R software packages. Created with Biorender.com.

All participants were categorized into three groups based on standardized arch index (AI) measurements (36, 37): low arch (LA, AI ≥ 0.26), normal arch (NA, 0.21 ≤ AI < 0.26), and high arch (HA, AI < 0.21). The AI was derived from plantar pressure testing, where the sole of the foot (excluding the toes) was divided into three equal areas, and the index was calculated as the midfoot-to-total pressure area ratio (38, 39). Within 24–48 h prior to testing, participants were required to avoid vigorous physical activity and ensure adequate sleep to achieve optimal physiological condition. All participants underwent plantar pressure testing and knee isokinetic muscle strength testing, conducted by separate evaluators. Data processing and analysis were performed using the R software package. The study design is illustrated in Figure 1.

### 2.2 Plantar pressure test

Static plantar pressure testing was performed using the Jasenco JSP-C5 Plantar Pressure Measurement and Analysis System (Beijing Jasenco Company). Key parameters included a resistive sensor type with a measurement capacity of >1,000,000, time accuracy of 0.005 s, and ≥1,600 sensors. Participants stood on the measurement plate with equal weight distribution, maintaining an upright posture, looking straight ahead, and naturally lowering their upper limbs for 5 s to capture static plantar pressure data. The system's integrated software analyzed the plantar area distribution to determine foot arch type (36). See Figure 1.

### 2.3 Isokinetic muscle strength test

An examiner blinded to group allocation used the ISOMED 2000 system (D&R Germany) to evaluate knee flexor-extensor muscle strength of the dominant leg [determined via kicking tests (40)]. Following a standardized 5-min cycle warm-up on a power bike, participants were securely positioned on the isokinetic device platform with trunk and thigh stabilization, ensuring proper alignment between the dynamometer's rotation axis and the knee's flexion-extension axis. The fixed testing protocol for all participants (41) consisted of 10 repetitions at 180 °/s, followed by 5 repetitions at 60 °/s (42), with a 1-min rest interval between the two velocities sets. Standardized verbal encouragement (Come on! push hard!) was provided during testing. Prior to each testing set, participants performed three practice repetitions of knee flexion-extension movements for familiarization. Extension and flexion moments were recorded throughout a 10°–90° knee range of motion. See Figure 1.

Angular moment curve data were captured at 5-ms intervals. Maximum moments were calculated at each angle (1°) (e.g., 20.1°–20.9°) based on muscle force definitions. These values were arranged according to the standard 10°–90°–10° test sequence. Missing or filtered data during acceleration/deceleration phases were interpolated twice, followed by data reversal to compute standardized moments at 1° increments, yielding a standardized moment profile with 1° resolution. Accelerated speed occurs only at the initiation of movement in each direction, typically lasting several tens or a few milliseconds. Moment data generated during these phases were excluded from the final analysis of isokinetic muscle strength (43).

### 2.4 Statistical analysis

Data were processed using SPSS 26.0 and the R software package (version 4.4.2). Normality was assessed using the Shapiro–Wilk test. Normally distributed data were reported as mean ± standard deviation. Homogeneity of variance among groups was assessed using Levene's test prior to between-group comparisons. For groups with homogeneous variances, one-way ANOVA was used for between-group comparisons, with *post-hoc* pairwise comparisons adjusted using the Bonferroni correction. For groups with heterogeneous variances, Welch's ANOVA was applied to account for unequal variances, followed by the Dunnett T3 method for pairwise comparisons. Non-normally distributed data were reported as median (interquartile range, IQR) and compared using the Kruskal–Wallis *H*-test, with multiple comparisons assessed using Dunn's test. Categorical data were presented as frequencies and percentages [(%)], with between-group comparisons performed using the R × C chi-square test. Isokinetic moment curves were characterized using generalized additive mixed models (GAMMs) fitted with a fully Bayesian approach via the brms package in R (44). The significance level was set at *α* = 0.05.

## 3 Results

### 3.1 Model information

The source code and a detailed summary of all models analyzing knee extension and flexion moments at angular velocities of 180 °/s and 60 °/s are provided as Supplementary Materials. In every model, the credible intervals for the standard deviations of the smooth coefficients were sufficiently far away from zero (refer to the “Smoothing Spline Hyperparameters” section in each model summary), which substantiates a non-linear relationship between force moment and range of motion. Furthermore, both intercepts and smooth terms differed considerably across subjects (refer to the “Multilevel Hyperparameters” section in each model summary).

### 3.2 Demographic information

Among the 60 subjects, 20 (10 males, 10 females) were identified in each of the LA, NA, and HA groups based on plantar pressure test results. There were no significant differences between groups in sex (*χ*^2^ = 0, *P* = 1), age (*H* = 2.342, *P* = 0.310), height (*F* = 0.372, *P* = 0.691), or weight (*F* = 2.015, *P* = 0.148) (Table 1).

**Table 1:** Demographic information of the three groups.

Indicators	LA (*n* = 20)	NA (*n* = 20)	HA (*n* = 20)	*χ*^2^/*H*/*F* value	*P* value
Sex (male: female)	10:10	10:10	10:10	0	1
Age (years)	21.50 (21.00, 23.75)	21.50 (21.00, 22.50)	21.00 (21.00, 22.00)	2.342	0.310
Height (m)	1.67 ± 0.075	1.67 ± 0.084	1.69 ± 0.080	0.372	0.691
Weight (kg)	65.60 ± 13.11	60.20 ± 9.22	58.95 ± 6.60	2.015	0.148

LA, low arch; NA, normal arch; HA, high arch.

Data are mean ± standard deviation or median (IQR).

### 3.3 Isokinetic knee extension moment at 60 °/s

**Figure 2:**
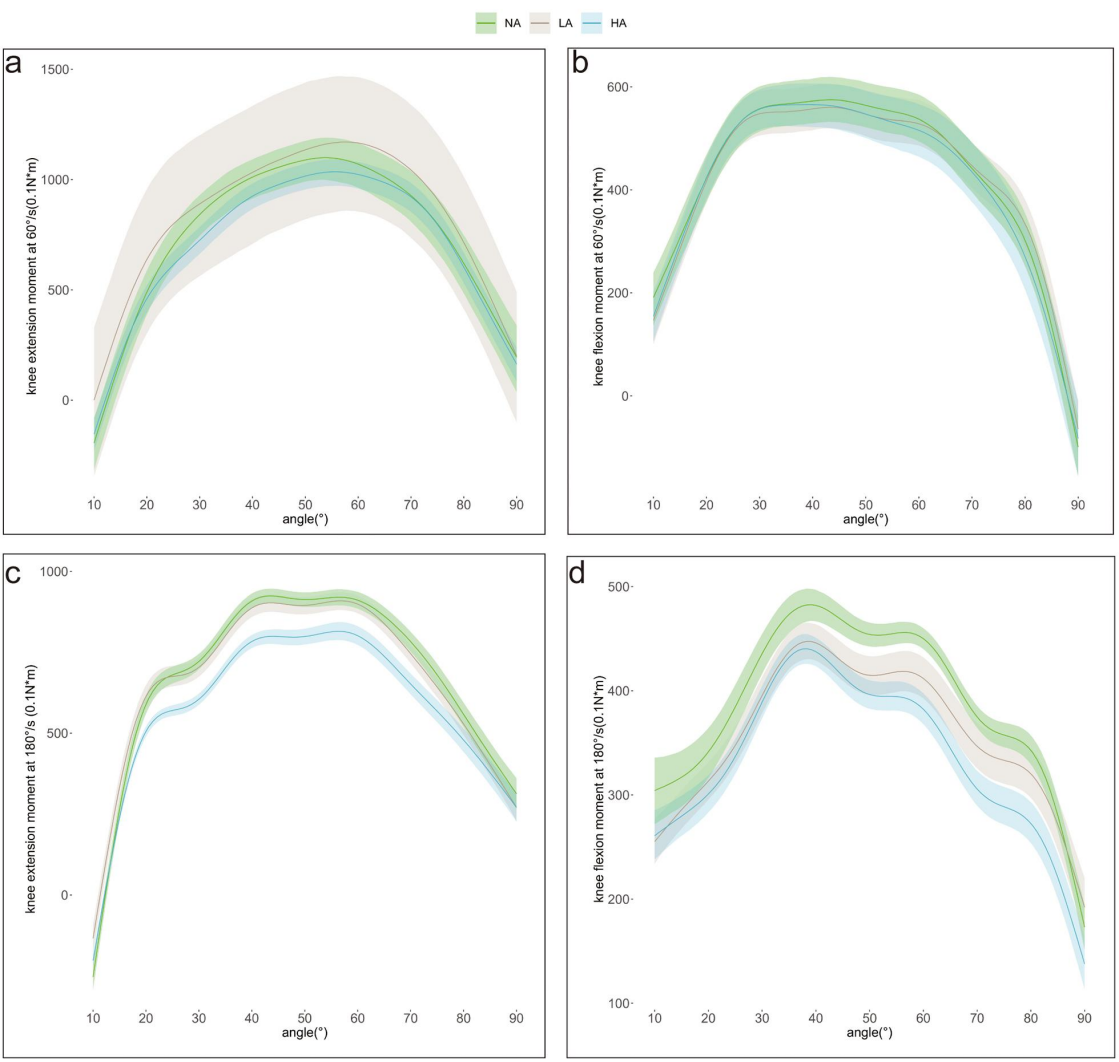
**(a,b)** (60 °/s) and **(c,d)** (180 °/s): isokinetic knee extension and flexion moments for the low arch (LA), high arch (HA) and normal arch (NA) groups. The shaded areas represent the credible interval (lower and upper bounds of the posterior distribution) for parameter estimation in Bayesian analysis.

Compared with the NA group, the LA group exhibited higher knee extension moments in the ranges of 10°–27°and 55°–83° (*P* < 0.05). Conversely, the HA group had lower knee extension moments in the ranges of 20°–66°and 82°–90°(*P* < 0.05), but significantly higher moments in the 10°–14°range (*P* < 0.05) (Figure 2a).

### 3.4 Isokinetic knee flexion moment at 60 °/s

Compared with the NA group, the LA group had lower knee flexion moments in the first half of the knee range of motion, with significant differences at 10°–17° and 31°–58° (*P* < 0.05); In the second half, the LA group had higher knee flexion moments, with a significant difference at 73°–90° (*P* < 0.05). The HA group had lower knee flexion moments than the NA group, with significant differences at 10°–15°, 43°–65°, and 74°–86° (*P* < 0.05) (Figure 2b).

### 3.5 Isokinetic knee extension moment at 180 °/s

Compared with the NA group, the LA group had lower knee extension moments at 26°–90° (*P* < 0.05), but higher moments at 10°–22° (*P* < 0.05).The HA group had lower knee extension moments at 14°–90° (*P* < 0.05), but higher moments at 10°–12°(*P* < 0.05) compared with the NA group (Figure 2c).

### 3.6 Isokinetic knee flexion moment at 180 °/s

Compared with the NA group, the LA group had lower knee flexion moments in the 10°–80° range (*P* < 0.05). Similarly, the HA group had lower knee flexion moments than the NA group in the 10°–90° range (*P* < 0.05) (Figure 2d).

## 4 Discussion

This study examined the IMC of the dominant lower limb among three arch height groups (LA, NA, HA) during maximal effort contractions at slow (60 °/s) and fast (180 °/s) angular velocities, focusing on knee extension and flexion moments at specific joint angles. At 60 °/s (slow velocity), LA individuals exhibited greater knee extension moments than NA controls, whereas HA individuals showed the opposite trend. Both LA and HA groups demonstrated reduced knee flexion moments relative to the NA group. Notably, at the terminal phase of joint movement, LA individuals displayed higher knee flexion moments than NA individuals. At 180 °/s (fast velocity), both LA and HA groups consistently exhibited weaker knee flexion and extension moments.

Moment values at the initiation and termination of joint motion are often compromised by isokinetic apparatus lever arm effects during acceleration and deceleration phases (45–47). To address this, we applied two-point interpolation to correct data. Empirical evidence indicates that moment data derived from this method are reliable within specific angular ranges: 6°–84° at 60 °/s and 20°–70° at 180 °/s (43). Deviations in IMC have been correlated strongly with knee joint function (31, 48). Therefore, changes in joint function can be promptly assessed by examining the morphology of IMC within these reliable angular intervals. Based on this premise, the present study analyzed the functional status of knee muscles within the established reliable angular ranges (48).

In isokinetic muscle strength analysis, moments recorded at slower velocities (60 °/s) are typically indicative of muscle strength (32). Our results revealed that at 60 °/s, individuals with LA exhibited higher knee extension moments than those with NA, whereas their knee flexion moments were comparatively lower. Notably, LA subjects demonstrated greater flexion moments than NA subjects only at the terminal phase of movement. This observation aligns with a study on ankle muscle strength, which similarly documented reduced strength in HA individuals and enhanced strength in LA subjects (22). However, previous studies have reported conflicting findings. Bakırhan et al. (24) conducted a cross-sectional study on 78 LA subjects and found no significant correlation between knee flexor and extensor strengths in LA and NA individuals. Similarly, Kim et al. (25) reported that although LA subjects exhibited greater knee abduction moments compared to NA subjects, there was no significant correlation in knee flexion and extension moments between the two groups. These discrepancies may stem from variations in the methods employed for moment measurement. Bakırhan et al. (24) used unarmed plyometrics, which is inherently subjective and constrained by its limitations. Kim et al. (25) measured ground reaction forces using a pressure plate and calculated moment magnitude indirectly by multiplying these forces by the force arm, a method limited by individual variations in force arm length. In contrast, our study employed an isokinetic muscle strength testing system, which allows for direct acquisition of moment data, thereby circumventing the limitations associated with the methodologies used by Bakırhan et al. and Kim et al. This direct measurement approach provides a more reliable and objective assessment of muscle strength and function, offering clearer insights into the biomechanical differences among the three groups.

The elevated knee extension moments observed in individuals with LA compared to those with NA may be attributed to two primary factors. First, it may represent a compensatory neuromuscular mechanism. LA individuals may require greater muscle strength to counteract knee abduction moments and maintain knee stability during daily activities. A meta-analysis (49) demonstrated that LA can increase knee abduction moments, which may necessitate higher knee adduction moments for balance and joint stability. Additionally, studies (50–52) have indicated that individuals with LA may experience impaired balance and joint position sense, further supporting the need for compensatory mechanisms. Second, this phenomenon may be related to the quadriceps angle (*Q* angle). Powers et al. (53) demonstrated that lower extremity alignment significantly affects patellar forces. The *Q* angle is formed by the intersection of the quadriceps force line and the line from the patellar center to the tibial tuberosity. External tibial rotation increases the *Q* angle, while internal rotation decreases it. A larger *Q* angle increases resistance to knee extension, reducing the knee extension moment. Conversely, individuals with internal knee rotation typically have smaller *Q* angles, which enhances the knee extension moment. Consequently, arch height variations lead to characteristic differences: LA (low arch) typically presents with a smaller *Q*-angle, while HA (high arch) demonstrates the opposite pattern. However, LA individuals exhibited lower knee flexion moments compared to NA individuals, likely due to altered gait characteristics. Marouvo et al. (4) observed greater peak knee flexion during gait in LA subjects, which may alter knee flexor contraction patterns and load distribution, potentially weakening these muscles over time.

Furthermore, in individuals with low arches, the subtalar and talonavicular joints exhibit excessive motion during the early to mid-stance phases of the gait cycle, and demonstrate a dysfunctional state in the late stance phase (54). This aberrant pattern further induces excessive foot pronation, which alters ankle kinematics and mechanical loading, thereby compromising knee joint function (55). Conversely, individuals with high arches, due to heightened foot rigidity and poor shock absorption, are prone to stress concentration along the lateral foot border (6, 7). During the gait cycle, the foot in these individuals often maintains a “locked” posture characterized by hindfoot varus and forefoot varus throughout the stance phase (3), which restricts effective stress dissipation and may predispose to fifth metatarsal stress fractures (56). Studies (57) have also indicated that high-arched athletes exhibit a higher incidence of bony structural injuries in the lateral lower limb, such as tibial and fifth metatarsal stress fractures, whereas low-arched athletes show a higher prevalence of medial soft tissue injuries, including patellar tendinopathy, Achilles tendinitis, and patellofemoral pain syndrome. These findings suggest that structural foot abnormalities can increase the risk of injuries at the ankle and knee by affecting the coordinated motion coupling mechanisms of the lower limb joints (58). Research demonstrates that personalized orthotic insoles can significantly alter plantar pressure distribution and bone stress (59, 60), indicating that future interventional studies could focus on customized footwear to explore its potential in alleviating proximal joint loads by improving foot biomechanics.

Our study found that knee extension and flexion moments were lower in HA individuals compared to NA individuals under slow-speed exercise conditions. This finding aligns with the work of Zhao et al. (61), who reported an inverse relationship between arch height and lower limb muscle strength in adult males, with HA individuals exhibiting diminished muscular performance. Williams et al. (7) demonstrated through electromyographic analysis that lateral femoral muscles in HA individuals activate earlier during exercise, indicating early knee extension activity. This early activation may increase limb stiffness upon foot contact with the ground (22). Additionally, Mao et al. (11) noted differences in anterior-posterior and internal-external ground reaction forces during the stance phase between HA and NA runners, which may be related to reduced lower limb muscle activity and altered muscle work. Thus, it is hypothesized that increased leg stiffness and reduced lower limb muscle activity may contribute to weaker knee musculature in HA individuals, potentially predisposing them to knee injuries.

Additionally, muscle strength performance in both LA and HA individuals is influenced by the *Q* angle. Powers et al. (53) demonstrated that normal lower extremity alignment is crucial for patellar outward force. A larger *Q* angle increases the lateral vector acting on the patella during knee extension, thereby augmenting the resistance to extension and reducing the knee extension moment. In HA individuals, external tibial rotation laterally displaces the tibial tuberosity, increasing the *Q* angle and the associated lateral vector, which in turn yields a smaller knee extension moment. Conversely, in LA individuals, internal tibial rotation medially shifts the tibial tuberosity, decreasing the *Q* angle and the lateral vector, thereby producing a larger knee extension moment. Accordingly, LA individuals typically exhibit greater knee extension strength than HA individuals.

In high-speed (180 °/s) sports, muscles primarily rely on the recruitment of fast-twitch muscle fibers (type II fibers) to generate force rapidly. These fibers are suited for short-duration, high-intensity activities such as sprinting and weightlifting (62, 63). In our study on the peak knee moments during rapid (180 °/s) motion, NA individuals exhibited the highest flexion and extension moments, followed by LA, with HA individuals showing the lowest values. This finding aligns with the work of Guenka et al. (23), who reported reduced isokinetic moment in the ankle joint among HA and LA individuals relative to NA individuals. Although their study focused on ankle musculature, the consistency in outcomes suggests a potential mechanistic link—specifically, that LA and HA individuals may possess a lower proportion of type II fibers. Reduced type II fiber content may decreases the muscle's capacity to produce maximal force in a short period, negatively impacting athletic performance (64, 65). Additionally, athletes with lower type II fiber content experience slower recovery after high-intensity exercise, increasing injury risk (66). To mitigate injury risk in LA and HA individuals during high-speed activities, several studies have emphasized the importance of targeted fast muscle resistance training to enhance the recruitment and function of fast-twitch fibers (63, 67, 68).

While the present study offers valuable insights into the relationship between arch height and knee isokinetic muscle strength at different angular velocities, several limitations should be acknowledged. First, the exclusive inclusion of young adults (20–30 years) may limit the extrapolation of findings to other age demographics, suggesting the need for broader age representation in future studies. Second, the singular focus on isokinetic muscle strength assessment precluded complementary evaluations of neuromuscular activation (via electromyography) and metabolic responses, which could offer more comprehensive mechanistic understanding. Third, the absence of participant exercise habits data represents an uncontrolled variable in our analysis. Fourth, the unilateral assessment protocol may constrain the generalizability of bilateral lower limb function inferences. Fifth, the use of a fixed testing order may introduce potential order effects. Sixth, although torque data from non-isokinetic phases were analytically excluded, the dynamometer may still struggle to maintain the preset speed perfectly within extremely short angular ranges, potentially leading to minor velocity fluctuations. Future studies could employ equipment with higher velocity-sampling precision or utilize velocity spectrum analysis to control this variable more rigorously. While our primary objective focused on examining knee flexion and extension muscle function across different foot arch types, subsequent research would benefit from incorporating bilateral measurements alongside integrated electrophysiological and metabolic assessments to more thoroughly characterize these functional relationships.

## 5 Conclusions

In summary, our study demonstrates that arch height is associated with knee muscle strength under varying exercise speeds. Specifically, at 60 °/s, individuals with LA exhibit greater muscle strength in the knee extension and the posterior half of the knee flexion range. Conversely, at 180 °/s, both LA and HA individuals display diminished knee muscle strength. These findings suggest that individuals with non-normal arch heights may benefit from incorporating targeted high-speed knee exercise training to enhance muscle performance and mitigate injury risk in competitive sports. Future studies will focus on the arch characteristics of patients with sports-related knee injuries and further explore the influence of arch height on knee function in this population.

## Data Availability

The original contributions presented in the study are included in the article/Supplementary Material, further inquiries can be directed to the corresponding authors.
