# Supplementary material for: Influence of foot arch height on isokinetic knee muscle strength at varying angular velocities
Source: Front Sports Act Living. 2026 Mar 24;8:1616379. doi: 10.3389/fspor.2026.1616379 (PMC13055593; doi:10.3389/fspor.2026.1616379)
Supplement: Supplementary file 1 [file Presentation1.pdf]

R code for the models of isokinetic strength curves using generalized additive multilevel models

```
#concentric knee extension at 180°/s in NA group

ke0_e180_ye <- brm(bf(torque ~ s(angle)+(angle|currentrepetition)),
                   data = kezu0_e180_ye,
                   family = gaussian(),
                   chains = 4,
                   control = list(adapt_delta = 0.95,max_treedepth = 15))
summary(ke0_e180_ye)
```

> summary(ke0_e180_ye)

```
 Family: gaussian
  Links: mu = identity; sigma = identity
Formula: torque ~ s(angle) + (angle | currentrepetition)
   Data: kezu0_e180_ye (Number of observations: 15633)
  Draws: 4 chains, each with iter = 2000; warmup = 1000; thin = 1;
         total post-warmup draws = 4000

Smoothing Spline Hyperparameters:
              Estimate Est.Error l-95% CI u-95% CI Rhat
sds(sangle_1)  1766.25    445.65  1137.89  2870.55 1.00
              Bulk_ESS Tail_ESS
sds(sangle_1)      748     1264

Multilevel Hyperparameters:
~currentrepetition (Number of levels: 10)
                      Estimate Est.Error l-95% CI u-95% CI
sd(Intercept)            59.48     17.57    34.71   104.21
sd(angle)                 1.24      0.36     0.74     2.05
cor(Intercept,angle)     -0.88      0.10    -0.98    -0.59
                      Rhat Bulk_ESS Tail_ESS
sd(Intercept)         1.00     1211     1862
sd(angle)             1.00     1121     1957
cor(Intercept,angle) 1.00     1364     1908

Regression Coefficients:
          Estimate Est.Error l-95% CI u-95% CI Rhat
Intercept   682.32      9.73   662.81   701.77 1.00
sangle_1   6167.93    286.83  5607.51  6738.78 1.00
          Bulk_ESS Tail_ESS
```

```
Intercept       1280     1469
sangle_1        2797     2701

Further Distributional Parameters:
      Estimate Est.Error l-95% CI u-95% CI Rhat Bulk_ESS
sigma   269.22      1.53   266.16   272.20 1.00     5279
      Tail_ESS
sigma     3152

Draws were sampled using sampling(NUTS). For each parameter, Bulk_ESS
and Tail_ESS are effective sample size measures, and Rhat is the potential
scale reduction factor on split chains (at convergence, Rhat = 1).

#concentric knee extension at 180°/s in LA group

ke1_e180_ye <- brm(bf(torque ~ s(angle)+(angle|currentrepetition)),
                   data = kezu1_e180_ye,
                   family = gaussian(),
                   chains = 4,
                   control = list(adapt_delta = 0.95,max_treedepth = 15))
summary(ke1_e180_ye)
```

> summary(ke1_e180_ye)

```
 Family: gaussian
  Links: mu = identity; sigma = identity
Formula: torque ~ s(angle) + (angle | currentrepetition)
   Data: kezu1_e180_ye (Number of observations: 14418)
  Draws: 4 chains, each with iter = 2000; warmup = 1000; thin = 1;
         total post-warmup draws = 4000

Smoothing Spline Hyperparameters:
              Estimate Est.Error l-95% CI u-95% CI Rhat
sds(sangle_1)  1730.66    437.60  1134.50  2774.53 1.01
              Bulk_ESS Tail_ESS
sds(sangle_1)      827     1170

Multilevel Hyperparameters:
~currentrepetition (Number of levels: 10)
                      Estimate Est.Error l-95% CI u-95% CI
sd(Intercept)            63.00     18.40    36.56   106.22
sd(angle)                 1.02      0.31     0.60     1.79
cor(Intercept,angle)     -0.83      0.13    -0.97    -0.47
                      Rhat Bulk_ESS Tail_ESS
```

```
sd(Intercept)              1.00      1058      1652
sd(angle)                  1.00       920      1769
cor(Intercept,angle) 1.00      1298      1880

Regression Coefficients:
             Estimate Est.Error l-95% CI u-95% CI Rhat
Intercept     672.89     11.93   647.98   696.70 1.00
sangle_1     5223.08    291.21  4641.81  5787.89 1.00
             Bulk_ESS Tail_ESS
Intercept        1231     1435
sangle_1         2713     2685

Further Distributional Parameters:
      Estimate Est.Error l-95% CI u-95% CI Rhat Bulk_ESS
sigma   259.55      1.53   256.54   262.58 1.00     5594
      Tail_ESS
sigma     2689
```

Draws were sampled using sampling(NUTS). For each parameter, Bulk_ESS and Tail_ESS are effective sample size measures, and Rhat is the potential scale reduction factor on split chains (at convergence, Rhat = 1).

#concentric knee extension at 180°/s in HA group

```
ke2_e180_ye <- brm(bf(torque ~ s(angle)+(angle|currentrepetition)),
                   data = kezu2_e180_ye,
                   family = gaussian(),
                   chains = 4,
                   control = list(adapt_delta = 0.95,max_treedepth = 15))
summary(ke2_e180_ye)
```

> summary(ke2_e180_ye)

```
 Family: gaussian
  Links: mu = identity; sigma = identity
Formula: torque ~ s(angle) + (angle | currentrepetition)
   Data: kezu2_e180_ye (Number of observations: 16119)
  Draws: 4 chains, each with iter = 2000; warmup = 1000; thin = 1;
         total post-warmup draws = 4000

Smoothing Spline Hyperparameters:
```

```
                 Estimate Est.Error l-95% CI u-95% CI Rhat
sds(sangle_1)   1598.18    402.98   1025.31   2555.27 1.00
                 Bulk_ESS Tail_ESS
sds(sangle_1)       854     1314
```

Multilevel Hyperparameters:

~currentrepetition (Number of levels: 10)

```
                        Estimate Est.Error l-95% CI u-95% CI
sd(Intercept)               6.37      5.55     0.24    19.96
sd(angle)                   0.65      0.21     0.36     1.16
cor(Intercept,angle)       -0.04      0.56    -0.94     0.94
                        Rhat Bulk_ESS Tail_ESS
sd(Intercept)           1.00     2545     2210
sd(angle)               1.00     1266     1982
cor(Intercept,angle) 1.01      377     1104
```

Regression Coefficients:

```
            Estimate Est.Error l-95% CI u-95% CI Rhat
Intercept     587.28     10.93   565.90   609.57 1.00
sangle_1     5181.76    282.42  4622.43  5749.90 1.00
            Bulk_ESS Tail_ESS
Intercept       1762     2115
sangle_1        3363     2836
```

Further Distributional Parameters:

```
      Estimate Est.Error l-95% CI u-95% CI Rhat Bulk_ESS
sigma   265.44      1.47   262.64   268.34 1.00     6961
      Tail_ESS
sigma     3330
```

Draws were sampled using sampling(NUTS). For each parameter, Bulk_ESS and Tail_ESS are effective sample size measures, and Rhat is the potential scale reduction factor on split chains (at convergence, Rhat = 1).

#concentric knee flexion at 180°/s in NA group

```
ke0_f180_ye <- brm(bf(torque ~ s(angle)+(angle|currentrepetition)),
                   data = kezu0_f180_ye,
                   family = gaussian(),
                   chains = 4,
                   control = list(adapt_delta = 0.95,max_treedepth = 15))
summary(ke0_f180_ye)
```

> summary(ke0_f180_ye)

```
 Family: gaussian
  Links: mu = identity; sigma = identity
Formula: torque ~ s(angle) + (angle | currentrepetition)
   Data: kezu0_f180_ye (Number of observations: 15633)
  Draws: 4 chains, each with iter = 2000; warmup = 1000; thin = 1;
         total post-warmup draws = 4000

Smoothing Spline Hyperparameters:
               Estimate Est.Error l-95% CI u-95% CI Rhat
sds(sangle_1)    511.09    132.12   316.19   832.24 1.00
               Bulk_ESS Tail_ESS
sds(sangle_1)       794     1103

Multilevel Hyperparameters:
~currentrepetition (Number of levels: 10)
                      Estimate Est.Error l-95% CI u-95% CI
sd(Intercept)            48.89     13.20    30.20    82.05
sd(angle)                 0.77      0.21     0.47     1.27
cor(Intercept,angle)     -0.98      0.03    -1.00    -0.91
                      Rhat Bulk_ESS Tail_ESS
sd(Intercept)         1.00     1037     1452
sd(angle)             1.00     1092     1604
cor(Intercept,angle) 1.00     1160     2088

Regression Coefficients:
          Estimate Est.Error l-95% CI u-95% CI Rhat
Intercept   390.58      4.69   381.30   399.81 1.00
sangle_1  -1492.90    184.06 -1870.47 -1129.20 1.00
          Bulk_ESS Tail_ESS
Intercept     1071     1507
sangle_1      2261     2639

Further Distributional Parameters:
      Estimate Est.Error l-95% CI u-95% CI Rhat Bulk_ESS
sigma   166.82      0.94   164.97   168.70 1.00     5837
      Tail_ESS
sigma     2737

Draws were sampled using sampling(NUTS). For each parameter, Bulk_ESS
and Tail_ESS are effective sample size measures, and Rhat is the potential
scale reduction factor on split chains (at convergence, Rhat = 1).
```

#concentric knee flexion at 180°/s in LA group

```
ke1_f180_ye <- brm(bf(torque ~ s(angle)+(angle|currentrepetition)),
                   data = kezu1_f180_ye,
                   family = gaussian(),
                   chains = 4,
                   control = list(adapt_delta = 0.95,max_treedepth = 15))
summary(ke1_f180_ye)
```

> summary(ke1_f180_ye)

```
 Family: gaussian
  Links: mu = identity; sigma = identity
Formula: torque ~ s(angle) + (angle | currentrepetition)
   Data: kezu1_f180_ye (Number of observations: 14256)
  Draws: 4 chains, each with iter = 2000; warmup = 1000; thin = 1;
         total post-warmup draws = 4000

Smoothing Spline Hyperparameters:
              Estimate Est.Error l-95% CI u-95% CI Rhat
sds(sangle_1)   449.29    112.21   281.46   720.85 1.00
              Bulk_ESS Tail_ESS
sds(sangle_1)     1251     1901

Multilevel Hyperparameters:
~currentrepetition (Number of levels: 10)
                      Estimate Est.Error l-95% CI u-95% CI
sd(Intercept)            28.50      8.94    16.16    49.88
sd(angle)                 0.48      0.16     0.26     0.87
cor(Intercept,angle)     -0.50      0.27    -0.88     0.16
                      Rhat Bulk_ESS Tail_ESS
sd(Intercept)         1.00     1547     2292
sd(angle)             1.00     1348     1959
cor(Intercept,angle) 1.00     1175     1696

Regression Coefficients:
          Estimate Est.Error l-95% CI u-95% CI Rhat
Intercept   359.76      8.56   342.69   377.82 1.00
sangle_1   -766.70    155.81 -1087.59  -466.76 1.00
          Bulk_ESS Tail_ESS
Intercept     1320     1747
sangle_1      2352     2438

Further Distributional Parameters:
```

```
          Estimate Est.Error l-95% CI u-95% CI Rhat Bulk_ESS
sigma     146.89      0.86   145.26   148.58 1.00     5712
          Tail_ESS
sigma         3100
```

Draws were sampled using sampling(NUTS). For each parameter, Bulk_ESS and Tail_ESS are effective sample size measures, and Rhat is the potential scale reduction factor on split chains (at convergence, Rhat = 1).

#concentric knee flexion at 180°/s in HA group

```
ke2_f180_ye <- brm(bf(torque ~ s(angle)+(angle|currentrepetition)),
                   data = kezu2_f180_ye,
                   family = gaussian(),
                   chains = 4,
                   control = list(adapt_delta = 0.95,max_treedepth = 15))
summary(ke2_f180_ye)
```

> summary(ke2_f180_ye)

```
 Family: gaussian
  Links: mu = identity; sigma = identity
Formula: torque ~ s(angle) + (angle | currentrepetition)
   Data: kezu2_f180_ye (Number of observations: 15876)
  Draws: 4 chains, each with iter = 2000; warmup = 1000; thin = 1;
         total post-warmup draws = 4000

Smoothing Spline Hyperparameters:
               Estimate Est.Error l-95% CI u-95% CI Rhat
sds(sangle_1)    457.46    115.30   289.27   731.54 1.00
               Bulk_ESS Tail_ESS
sds(sangle_1)      1043     1851

Multilevel Hyperparameters:
~currentrepetition (Number of levels: 10)
                      Estimate Est.Error l-95% CI u-95% CI
sd(Intercept)            36.58     10.85    21.36    63.42
sd(angle)                 0.67      0.20     0.40     1.16
cor(Intercept,angle)     -0.86      0.12    -0.98    -0.55
                      Rhat Bulk_ESS Tail_ESS
sd(Intercept)         1.00     1235     1812
sd(angle)             1.00     1164     1708
cor(Intercept,angle) 1.00     1390     2139
```

```
Regression Coefficients:
          Estimate Est.Error l-95% CI u-95% CI Rhat
Intercept   336.08      6.16   323.98   348.27 1.00
sangle_1  -1001.40    151.01 -1294.69  -713.37 1.00
          Bulk_ESS Tail_ESS
Intercept     1397     2048
sangle_1      2027     2525

Further Distributional Parameters:
      Estimate Est.Error l-95% CI u-95% CI Rhat Bulk_ESS
sigma   145.19      0.82   143.61   146.79 1.00     4650
      Tail_ESS
sigma     2760

Draws were sampled using sampling(NUTS). For each parameter, Bulk_ESS
and Tail_ESS are effective sample size measures, and Rhat is the potential
scale reduction factor on split chains (at convergence, Rhat = 1).
```

#concentric knee extension at 60°/s in NA group

```
ke0_e60_ye <- brm(bf(torque ~ s(angle)+(angle|currentrepetition)),
                  data = kezu0_e60_ye,
                  family = gaussian(),
                  chains = 4,
                  control = list(adapt_delta = 0.95,max_treedepth = 15))
summary(ke0_e60_ye)
```

> summary(ke0_e60_ye)

```
 Family: gaussian
  Links: mu = identity; sigma = identity
Formula: torque ~ s(angle) + (angle | currentrepetition)
   Data: kezu0_e60_ye (Number of observations: 7857)
  Draws: 4 chains, each with iter = 2000; warmup = 1000; thin = 1;
         total post-warmup draws = 4000

Smoothing Spline Hyperparameters:
              Estimate Est.Error l-95% CI u-95% CI Rhat
sds(sangle_1)   976.27    248.77   622.04  1584.22 1.00
              Bulk_ESS Tail_ESS
sds(sangle_1)     1236     1901

Multilevel Hyperparameters:
~currentrepetition (Number of levels: 5)
```

```
                              Estimate Est.Error l-95% CI u-95% CI
sd(Intercept)                   128.97     66.93    57.16   301.00
sd(angle)                         2.32      1.15     1.03     5.57
cor(Intercept,angle)             -0.78      0.27    -0.99     0.03
                              Rhat Bulk_ESS Tail_ESS
sd(Intercept)                 1.00     1241     1650
sd(angle)                     1.00     1191     1546
cor(Intercept,angle) 1.00     1197     1738
```

Regression Coefficients:

```
          Estimate Est.Error l-95% CI u-95% CI Rhat
Intercept   756.99     48.67   660.75   843.83 1.00
sangle_1   2409.53    485.63  1450.84  3347.51 1.00
          Bulk_ESS Tail_ESS
Intercept     1185      880
sangle_1      2478     2449
```

Further Distributional Parameters:

```
      Estimate Est.Error l-95% CI u-95% CI Rhat Bulk_ESS
sigma   339.08      2.67   333.78   344.26 1.00     5299
      Tail_ESS
sigma     2637
```

Draws were sampled using sampling(NUTS). For each parameter, Bulk_ESS and Tail_ESS are effective sample size measures, and Rhat is the potential scale reduction factor on split chains (at convergence, Rhat = 1).

```
#concentric knee extension at 60°/s in LA group

ke1_e60_ye <- brm(bf(torque ~ s(angle)+(angle|currentrepetition)),
                  data = kezu1_e60_ye,
                  family = gaussian(),
                  chains = 4,
                  control = list(adapt_delta = 0.95,max_treedepth = 15))
summary(ke1_e60_ye)
```

> summary(ke1_e60_ye)

```
 Family: gaussian
  Links: mu = identity; sigma = identity
Formula: torque ~ s(angle) + (angle | currentrepetition)
   Data: kezu1_e60_ye (Number of observations: 7938)
  Draws: 4 chains, each with iter = 2000; warmup = 1000; thin = 1;
         total post-warmup draws = 4000
```

```
Smoothing Spline Hyperparameters:
                     Estimate Est.Error l-95% CI u-95% CI Rhat
sds(sangle_1)    1103.58       270.92      707.64    1772.57 1.00
                     Bulk_ESS Tail_ESS
sds(sangle_1)         1524         2430

Multilevel Hyperparameters:
~currentrepetition (Number of levels: 10)
                               Estimate Est.Error l-95% CI u-95% CI
sd(Intercept)                  583.85       142.42      379.31      931.95
sd(angle)                           3.14           1.02         1.70         5.66
cor(Intercept,angle)       -0.50           0.26       -0.87         0.11
                               Rhat Bulk_ESS Tail_ESS
sd(Intercept)              1.00         1222         1988
sd(angle)                     1.00         1331         2133
cor(Intercept,angle) 1.00         2441         2619

Regression Coefficients:
               Estimate Est.Error l-95% CI u-95% CI Rhat
Intercept      842.76       154.19      529.88    1144.20 1.00
sangle_1      1437.25       438.03      576.21    2296.98 1.00
               Bulk_ESS Tail_ESS
Intercept        1058         1657
sangle_1          2718         2671

Further Distributional Parameters:
          Estimate Est.Error l-95% CI u-95% CI Rhat Bulk_ESS
sigma     296.93          2.35      292.27      301.71 1.00         6005
          Tail_ESS
sigma        2895

Draws were sampled using sampling(NUTS). For each parameter, Bulk_ESS
and Tail_ESS are effective sample size measures, and Rhat is the potential
scale reduction factor on split chains (at convergence, Rhat = 1).

#concentric knee extension at 60°/s in HA group

ke2_e60_ye <- brm(bf(torque ~ s(angle)+(angle|currentrepetition)),
                         data = kezu2_e60_ye,
                         family = gaussian(),
                         chains = 4,
                         control = list(adapt_delta = 0.95,max_treedepth = 15))
summary(ke2_e60_ye)
```

> summary(ke2_e60_ye)

```
 Family: gaussian
  Links: mu = identity; sigma = identity
Formula: torque ~ s(angle) + (angle | currentrepetition)
   Data: kezu2_e60_ye (Number of observations: 7857)
  Draws: 4 chains, each with iter = 2000; warmup = 1000; thin = 1;
         total post-warmup draws = 4000

Smoothing Spline Hyperparameters:
              Estimate Est.Error l-95% CI u-95% CI Rhat
sds(sangle_1)  1022.22    249.31   657.10  1615.47 1.00
              Bulk_ESS Tail_ESS
sds(sangle_1)     1163     1729

Multilevel Hyperparameters:
~currentrepetition (Number of levels: 5)
                     Estimate Est.Error l-95% CI u-95% CI
sd(Intercept)           71.34     37.41    29.19   167.44
sd(angle)                0.94      0.57     0.31     2.39
cor(Intercept,angle)    -0.65      0.35    -0.99     0.31
                     Rhat Bulk_ESS Tail_ESS
sd(Intercept)        1.00     1074     1650
sd(angle)            1.00     1273     1790
cor(Intercept,angle) 1.00     1770     2303

Regression Coefficients:
          Estimate Est.Error l-95% CI u-95% CI Rhat
Intercept   712.35     27.46   652.65   766.85 1.00
sangle_1   2137.63    420.31  1314.36  2971.40 1.00
          Bulk_ESS Tail_ESS
Intercept     1594     1526
sangle_1      2624     2184

Further Distributional Parameters:
      Estimate Est.Error l-95% CI u-95% CI Rhat Bulk_ESS
sigma   286.27      2.27   281.92   290.79 1.00     5531
      Tail_ESS
sigma     2945

Draws were sampled using sampling(NUTS). For each parameter, Bulk_ESS
and Tail_ESS are effective sample size measures, and Rhat is the potential
scale reduction factor on split chains (at convergence, Rhat = 1).
```

#concentric knee flexion at 60°/s in NA group

```
ke0_f60_ye <- brm(bf(torque ~ s(angle)+(angle|currentrepetition)),
                  data = kezu0_f60_ye,
                  family = gaussian(),
                  chains = 4,
                  control = list(adapt_delta = 0.95,max_treedepth = 15))
summary(ke0_f60_ye)
```

> summary(ke0_f60_ye)

```
 Family: gaussian
  Links: mu = identity; sigma = identity
Formula: torque ~ s(angle) + (angle | currentrepetition)
   Data: kezu0_f60_ye (Number of observations: 7857)
  Draws: 4 chains, each with iter = 2000; warmup = 1000; thin = 1;
         total post-warmup draws = 4000

Smoothing Spline Hyperparameters:
              Estimate Est.Error l-95% CI u-95% CI Rhat
sds(sangle_1)   746.84    197.56   458.14  1238.71 1.00
              Bulk_ESS Tail_ESS
sds(sangle_1)     1309     2017

Multilevel Hyperparameters:
~currentrepetition (Number of levels: 5)
                      Estimate Est.Error l-95% CI u-95% CI
sd(Intercept)            45.26     28.27    14.69   120.06
sd(angle)                 0.62      0.47     0.09     1.80
cor(Intercept,angle)     -0.52      0.42    -0.98     0.56
                      Rhat Bulk_ESS Tail_ESS
sd(Intercept)         1.00     1182     1645
sd(angle)             1.00      970      953
cor(Intercept,angle) 1.00     1808     1975

Regression Coefficients:
          Estimate Est.Error l-95% CI u-95% CI Rhat
Intercept   433.01     20.06   392.45   476.55 1.00
sangle_1  -1868.13    349.61 -2567.69 -1208.38 1.00
          Bulk_ESS Tail_ESS
Intercept     1340     1433
sangle_1      2295     2774
```

```
Further Distributional Parameters:
      Estimate Est.Error l-95% CI u-95% CI Rhat Bulk_ESS
sigma   239.82      1.86   236.18   243.43 1.00     5374
      Tail_ESS
sigma     3219

Draws were sampled using sampling(NUTS). For each parameter, Bulk_ESS
and Tail_ESS are effective sample size measures, and Rhat is the potential
scale reduction factor on split chains (at convergence, Rhat = 1).
```

#concentric knee flexion at 60°/s in LA group

```
ke1_f60_ye <- brm(bf(torque ~ s(angle)+(angle|currentrepetition)),
                  data = kezu1_f60_ye,
                  family = gaussian(),
                  chains = 4,
                  control = list(adapt_delta = 0.95,max_treedepth = 15))
summary(ke1_f60_ye)
```

> summary(ke1_f60_ye)

```
 Family: gaussian
  Links: mu = identity; sigma = identity
Formula: torque ~ s(angle) + (angle | currentrepetition)
   Data: kezu1_f60_ye (Number of observations: 7290)
  Draws: 4 chains, each with iter = 2000; warmup = 1000; thin = 1;
         total post-warmup draws = 4000

Smoothing Spline Hyperparameters:
              Estimate Est.Error l-95% CI u-95% CI Rhat
sds(sangle_1)   838.81    225.92   511.56  1371.12 1.00
              Bulk_ESS Tail_ESS
sds(sangle_1)     1281     1949

Multilevel Hyperparameters:
~currentrepetition (Number of levels: 5)
                      Estimate Est.Error l-95% CI u-95% CI
sd(Intercept)            39.00     24.70    13.69   101.61
sd(angle)                 0.16      0.17     0.01     0.61
cor(Intercept,angle)     -0.00      0.59    -0.96     0.95
                      Rhat Bulk_ESS Tail_ESS
sd(Intercept)         1.00     1462     1640
sd(angle)             1.00     2525     2288
cor(Intercept,angle)  1.00     4088     2611
```

```
Regression Coefficients:
            Estimate Est.Error l-95% CI u-95% CI Rhat
Intercept     430.81     20.43   390.00   474.07 1.00
sangle_1    -1607.41    322.85 -2230.09  -950.55 1.00
            Bulk_ESS Tail_ESS
Intercept       1480     1674
sangle_1        2364     2448

Further Distributional Parameters:
      Estimate Est.Error l-95% CI u-95% CI Rhat Bulk_ESS
sigma   212.46      1.75   209.02   215.86 1.00     5988
      Tail_ESS
sigma     2869

Draws were sampled using sampling(NUTS). For each parameter, Bulk_ESS
and Tail_ESS are effective sample size measures, and Rhat is the potential
scale reduction factor on split chains (at convergence, Rhat = 1).

#concentric knee flexion at 60°/s in HA group

ke2_f60_ye <- brm(bf(torque ~ s(angle)+(angle|currentrepetition)),
                  data = kezu2_f60_ye,
                  family = gaussian(),
                  chains = 4,
                  control = list(adapt_delta = 0.95,max_treedepth = 15))
summary(ke2_f60_ye)
```

> summary(ke2_f60_ye)

```
 Family: gaussian
  Links: mu = identity; sigma = identity
Formula: torque ~ s(angle) + (angle | currentrepetition)
   Data: kezu2_f60_ye (Number of observations: 7776)
  Draws: 4 chains, each with iter = 2000; warmup = 1000; thin = 1;
         total post-warmup draws = 4000

Smoothing Spline Hyperparameters:
              Estimate Est.Error l-95% CI u-95% CI Rhat
sds(sangle_1)   603.12    150.33   380.64   972.16 1.00
              Bulk_ESS Tail_ESS
sds(sangle_1)     1384     2267

Multilevel Hyperparameters:
```

~currentrepetition (Number of levels: 5)

	Estimate	Est.Error	l-95% CI	u-95% CI
sd(Intercept)	43.02	25.73	15.14	111.26
sd(angle)	0.92	0.65	0.34	2.53
cor(Intercept,angle)	-0.54	0.39	-0.97	0.47

	Rhat	Bulk_ESS	Tail_ESS
sd(Intercept)	1.01	1266	1529
sd(angle)	1.00	1158	1259
cor(Intercept,angle)	1.00	1420	1462

Regression Coefficients:

	Estimate	Est.Error	l-95% CI	u-95% CI	Rhat
Intercept	421.25	22.57	375.14	465.11	1.00
sangle_1	-889.84	297.90	-1501.90	-323.11	1.00

	Bulk_ESS	Tail_ESS
Intercept	1335	1283
sangle_1	2196	2083

Further Distributional Parameters:

	Estimate	Est.Error	l-95% CI	u-95% CI	Rhat	Bulk_ESS
sigma	215.19	1.72	211.91	218.56	1.00	5794

	Tail_ESS
sigma	2657

Draws were sampled using sampling(NUTS). For each parameter, Bulk_ESS and Tail_ESS are effective sample size measures, and Rhat is the potential scale reduction factor on split chains (at convergence, Rhat = 1).
